# Preliminary trial findings from Harvest Share: a cost-offset community supported agriculture model to improve diet in New York City

**DOI:** 10.1186/s12966-026-01906-z

**Published:** 2026-07-11

**Authors:** Madison N. LeCroy, Luisa Cardenas, Sze Wan Celine Chan, Maya Scherer, Brett Dolotina, Dalila Victoria Lanza, Rhyden Dowd, Michelle Hughes, Steve Mei, Kathleen Barth, Mayssa Gregoire, Ana I. Rodriguez Gil, Marisa DeDominicis, Maria Acevedo, Alina Rodriguez, Lorena Kourousias, Mary Cheng, Mitchel Wu, Jairo Guzman, Hayley M. Belli, R. Gabriela Barajas-Gonzalez, Lan N. Đoàn, Stella S. Yi

**Affiliations:** 1 Department of Population Health, NYU Grossman School of Medicine, 180 Madison Ave, 8th Floor, New York, NY 10016 USA; 2 Center for Evaluation and Applied Research, New York Academy of Medicine, 1216 5th Avenue, New York, NY 10029 USA; 3 Brooklyn Grange, 850 3rd Avenue, Brooklyn, NY 11232 USA; 4 Glynwood Regional Center for Food and Farming, 362 Glynnwood Road, Cold Spring, NY 10516 USA; 5 Chinese American Planning Council, 45 Suffolk Street, New York, NY 10002 USA; 6 The Table at the Family Health Centers at NYU Langone, 6025 6th Avenue, Brooklyn, NY 11220 USA; 7 Angel Family Farm, 6 Harvest Lane, Goshen, NY 10924 USA; 8 Earth Matter, 758 Enright Road, New York, NY 10004 USA; 9 Hot Bread Kitchen, 75 9th Avenue, Suite 0610, New York, NY 10011 USA; 10 Public School 169, 4305 7th Avenue, Brooklyn, NY 11232 USA; 11 Mixteca Organization, Inc., 245 23rd Street, 2nd Floor, Brooklyn, NY 11215 USA; 12 Mexican Coalition for the Empowerment of Youth and Families, Inc., 389 E 150th Street, Bronx, NY 10455 USA

**Keywords:** Diet, Community-based participatory research, Clinical trial, Community-supported agriculture

## Abstract

**Background:**

Community-supported agriculture (CSA) programs are an evidence-based approach to increase fruit and vegetable (F&V) intake while also strengthening the local farming economy and social bonds. However, uptake has been limited due to payment structures and/or CSA offerings not reflecting local community preferences. To address this need, we developed the Harvest Share program, which centers a cost-offset CSA tailored to local communities in New York City. Here, our objective is to conduct a mixed-methods evaluation of year one of Harvest Share to evaluate its preliminary impact on diet and social outcomes.

**Methods:**

Harvest Share participants (*n* = 542; *n* = 153 CSA and *n* = 389 comparison community [CC] participants) completed surveys and Veggie Meter readings—an objective measure of F&V intake—at the start and end of the 20-week growing season (~ June-October 2023). Qualitative interviews were conducted with *n* = 23 participants to understand program experiences. Mixed effect linear regression models were used to examine the impact of Harvest Share on primary (Veggie Meter score) and secondary outcomes (diet and social variables). Significant associations were based on the group (CSA vs. CC) by time (pre vs. post) interaction term. Descriptive, thematic analysis was used to analyze interviews.

**Results:**

Across CSA and CC participants, individuals were, on average, 44 years old; female (80%); Asian (48%) or Hispanic/Latino (37%); and of lower socioeconomic status (55% with annual household income <$50,000). Change in Veggie Meter scores (*p* = 0.02), F&V consumption (*p* < 0.0001), and F&V knowledge (*p* = 0.0004) significantly differed between CSA and CC participants. Veggie Meter scores trended downwards in both groups, but the decline was non-significant among CSA participants (CSA: -7.43 [95%CI: -21.30, 6.44]; CC: -27.29 [95%CI: -36.27, -18.30]). CSA participation was associated with an increased number of different types of F&V consumed (CSA: 3.74 [95%CI: 2.94, 4.53]; CC: 0.05 [95%CI: -0.48, 0.57]) and decreased number of F&V that individuals did *not* know how to prepare (CSA: -1.60 [95%CI: -2.28, -0.93]; CC: -0.13 [95%CI: -0.57, 0.32]). Qualitative interviews indicated the CSA also promoted family and community connection and improved physical and mental health.

**Conclusions:**

A cost-offset, tailored CSA can improve diet and social outcomes using a model that supports farmers.

**Trial registration:**

Registered on ClinicalTrials.gov as NCT05381766 (registration date: May 19, 2022).

**Supplementary Information:**

The online version contains supplementary material available at 10.1186/s12966-026-01906-z.

## Background

The 2020–2025 Dietary Guidelines for Americans recommend individuals consume 2½ cups of vegetables and 2 cups of fruit per day. However, almost 90% of the United States’ (US) population is not meeting vegetable recommendations, and ~ 80% of the population is not meeting fruit recommendations [1]. These percentages vary across socioeconomic backgrounds (e.g., 7% of individuals with a poverty-to-income ratio ≤1.85 vs. 15% of individuals with a poverty-to-income ratio >1.85 meet vegetable intake recommendations) and racial and ethnic groups (e.g., 12% of non-Hispanic White individuals, 5% of non-Hispanic Black, 21% of non-Hispanic Asian, and 12% of Hispanic individuals meet vegetable intake recommendations), with a clear need to improve intake across multiple, intersecting sociodemographic groups [2]. Adequate intake of fruits and vegetables (F&V) is a critical part of a healthy diet and has been associated with decreased risk for cardiometabolic disease [3–5]. Thus, there is a need to promote increased produce intake among the US population, particularly among individuals with elevated risk for cardiometabolic disease, including persons from lower socioeconomic status and among communities of color [6–9].

One evidence-based strategy for increasing the amount and variety of F&V individuals consume is community-supported agriculture (CSA) programs [10–15]. CSA is a model where individuals purchase a portion of a farm’s anticipated production at the beginning of the growing season in exchange for receiving shares (often weekly) throughout the growing season. Despite their benefits, participation in CSA programs has largely been limited to White females who are highly educated [10]. Factors including accessibility, lack of awareness of the program or CSA model, cost, and incongruence of produce offerings with community preferences have been barriers to participation for individuals of lower income and communities of color [16, 17].

To address financial barriers, there has been a recent rise in cost-offset CSAs, which provide subsidized shares and/or accept flexible payments (e.g., weekly payments vs. one-time upfront payment) [11, 12, 14, 15, 18–22]. Findings generally suggest that cost-offset CSAs and integrated programming can improve vegetable intake [11, 14, 18] and other determinants of physical and mental health, such as self-efficacy, nutrition knowledge, and social connection [13, 20, 23]. However, previous studies have been limited in being inclusive of all communities. Specifically, only four previous cost-offset CSA programs support enrollment for languages other than English [12, 20, 21, 24], and only three additional studies recruited samples where at least 25% of the sample identifies with a community of color [13, 19, 24]. Related formative and qualitative work for these studies also reflects this demographic [25, 26]. Notably, our previous pilot study is the only cost-offset CSA to (1) serve a primarily Asian American population, (2) provide Mandarin and Cantonese language support, and (3) tailor produce offerings to address a key participation barrier—mismatch in produce offerings and preferences [24]. By providing produce that aligns with community preferences, there is an opportunity to promote individuals’ sense of belonging and highlight cultural foods that may not be readily available for purchase elsewhere [27]. There is thus an opportunity to reach a broader audience and address diet-related disparities through the development and evaluation of a cost-offset CSA tailored to community preferences.

To address these gaps and respond to community needs, we developed Harvest Share, a whole-of-community intervention that centers a cost-offset CSA program featuring Mexican and Chinese produce and resources and support in multiple languages for residents living in Sunset Park—a neighborhood in New York City (NYC; Brooklyn) comprised primarily of Mexican and Chinese American families that was hard-hit during the COVID-19 pandemic, particularly in the food retail environment [28–30]. The aim of the present study was to assess the effectiveness of Harvest Share for improving F&V intake (primary outcome) and nutrition knowledge, sense of belonging, and related social measures (secondary outcomes). In this analysis, we present preliminary findings from a mixed-methods evaluation conducted during the first year of the program.

## Methods

Harvest Share is a multi-sectoral collaboration between academia, community-based organizations, local elementary schools, and local farms/gardens that launched in September 2022 to improve diet and food access in Sunset Park. The program builds on our previous pilot work where we demonstrated the feasibility, acceptability, and preliminary impacts of a cost-offset, CSA program tailored to community preferences [24]. The Harvest Share program has five pillars: (1) a cost-offset, CSA tailored to community preferences—thus, centering Chinese and Mexican produce—, (2) nutrition education, (3) experiential learning (e.g., farm tours, cooking demonstrations, gardening workshops), (4) policy (e.g., assessing local food policy), and (5) economic security (e.g., SNAP/WIC enrollment, local small business engagement).

Importantly, the Harvest Share program was tailored to the Sunset Park, Brooklyn community, where the community is predominantly immigrant Asian or Hispanic/Latino-identifying individuals (35% Asian [of which 90% identify as Chinese], 41% Hispanic/Latino [of which 42% identify as Mexican]; 47% born outside of the US), that faced socioeconomic setbacks as a result of the COVID-19 pandemic [28–30]. Of note, the Sunset Park community is geographically divided—the Hispanic/Latino community is concentrated along 5th Avenue and the Asian community along 8th Avenue [31]. To promote community cohesion, Harvest Share aimed to create a shared space where individuals of all backgrounds could comfortably interact. By rooting Harvest Share in community-based participatory research, we were able to develop programming and nutrition initiatives that uniquely honored distinct preferences while also promoting cross-cultural learning and community building. An overview of the tailored, cost-offset CSA and related programming (i.e., Pillars 1–3) is provided here. The Harvest Share program was approved by the NYU Langone Health IRB and is registered on ClinicalTrials.gov as NCT05381766 (registration date: May 19, 2022).

### CSA program overview

The Harvest Share CSA includes four 20-week growing seasons, but the present paper focuses only on Growing Season 1 (GS1; June-October 2023). Harvest Share partners with two farms for the CSA: Brooklyn Grange (BG), which grows the Chinese produce, and Angel Family Farm (AFF), which grows the Mexican produce. Participants selected which CSA to participate in during online registration. BG’s CSA was valued at $25/weekly share (participant cost: $7; total GS1 subsidy: $360), and the AFF CSA was valued at $29.75/weekly share (participant cost: $11; total GS1 subsidy: $375). During GS1, each BG CSA share included ~4 pounds of produce, and each AFF CSA share included ~5 pounds of produce. Boxes were not adjusted to household size. An example CSA share was as follows. For BG, one week included: napa cabbage (2 heads), A-choy (1 bunch), green/purple sprouting broccoli (1 bunch), pak choi (1 bunch), pea shoots (1 clamshell container), and garlic chives (1 bunch). For AFF, one week included: quelites (1 bunch), papalo (1 bunch), calabacito (1 unit), green beans (1 bag), basil (1 bunch), and cucumbers (3 pieces). Participants who received SNAP or WIC benefits could use their electronic benefit transfer (EBT) card to cover their CSA cost weekly. Produce offerings in each CSA were tailored to community preferences based on brief surveys amongst the community and semi-structured interviews [26].

Produce box pick-ups occurred weekly (Tuesdays from 3-7pm) at the BG rooftop farm in Sunset Park. Vegetables were displayed market-style on tables, and individuals selected produce for their share following bilingual signage (e.g., choose 1 bunch of bok choy, 3 heads of garlic). Individuals were guided to choose exactly one unit of each available vegetable option. Participants could choose not to take any of the vegetables, but if they did so, they were not able to select multiple units of the other types. If participants could not attend, friends and family were able to pick up their CSA share for them. All pick-ups were staffed by trained bilingual (English, Cantonese, Mandarin, Spanish) Harvest Share staff and volunteers. During the GS1 period, participants who requested to unenroll or who missed 2 or more pick-ups without alerting the Harvest Share team were removed from the study (*n* = 12 for BG and *n* = 6 for AFF). New participants were enrolled into the program to fill vacant slots until Week 10 for BG and Week 12 for AFF, with a maximum of 100 enrollees in BG and 40 enrollees in AFF at a time.

### Nutrition education and experiential learning

Throughout the 20-week GS1, nutrition education was offered in the following forms: in-person (during pick-up at the BG rooftop farm) or online webinars led by bilingual Harvest Share staff; in-person cooking tutorials by bilingual Harvest Share staff/volunteers and Harvest Share partners; and in-person gardening tutorials by BG and Earth Matter, a Harvest Share partner. Participants were also given a healthy, tailored recipe each week, in their preferred language, related to that week’s CSA produce offerings. In GS1, we hosted 6 nutrition education sessions (*n* = 81 participants), 4 cooking workshops (*n* = 69), 3 farming/gardening workshops (*n* = 45), 2 farm tours (*n* = 30), 1 physical activity session (*n* = 23), and 4 arts sessions for participants’ children (*n* = 57).

### Sample selection

To recruit participants for the Harvest Share CSA, the study team collaborated with the two farms, three local community-based organizations serving Chinese and Mexican families (Chinese-American Planning Council [CPC], Mixteca, Hot Bread Kitchen), The Table (a client-choice food pantry located in an NYU-affiliated federally qualified health center), and one public elementary school (P.S. 169). Recruitment consisted of the trained bilingual study team members attending in-person events at partner sites and partners distributing program flyers within their networks. Participants from our previous pilot study [24] were also invited to participate in Harvest Share. Individuals were eligible to participate if they 1) were ≥18 years of age; 2) lived in or around Sunset Park; and 3) were able to speak English, Spanish, Cantonese, Mandarin, or Bangla. To enroll in Harvest Share, interested and eligible participants completed an online registration form for their desired CSA program and reviewed the informed consent form. Bilingual study staff provided in-language remote support for form completion and any questions related to the program or consent form. After registering, the NYU study team reached out with instructions for the first produce box pick-up and a link to the baseline survey (see *Survey* section). Participants signed a physical consent form during the first produce pick-up for GS1.

Although recruitment materials were made available to individuals who speak Bangla, no participants who spoke Bangla were recruited for GS1. Thus, Harvest Share programming tailored to Bangla-speakers was developed in parallel to GS1 and will be described in a future manuscript.

### CSA program evaluation

Harvest Share CSA participants completed a survey and Veggie Meter assessment at the time of enrollment and again at the end of the 20-week growing season (June-October 2023). During approximately the same calendar months (June-August and October-November 2023), we also conducted a pre-post evaluation among “comparison communities” in NYC chosen for their large Chinese (Chinatown, Manhattan and Flushing, Queens) and Mexican (Mott Haven, South Bronx) communities. Individuals in these communities were recruited using a similar approach as described for the CSA and were eligible if they 1) were ≥18 years of age; 2) lived in and around 1 of the 3 target communities; and 3) were able to speak English, Spanish (Mott Haven in the South Bronx), Cantonese (Chinatown and Flushing), or Mandarin (Chinatown and Flushing). The recruitment goals for the comparison communities were: *n* = 100 in Chinatown, *n* = 100 in Flushing, and *n* = 200 in the South Bronx. After confirming eligibility and consenting, individuals received a link to the baseline survey (see *Survey* section) and information on when to complete their Veggie Meter reading in-person.

The Harvest Share CSA program is evaluated using a pre-post non-equivalent control group design, with the comparison communities as our control group [32]. Our primary outcome was the pre-post change in objectively measured F&V intake, as assessed by the Veggie Meter, for CSA participants vs. comparison community members. Secondary outcomes, including changes in self-reported dietary intake and other health and sociocultural measures, were evaluated using surveys.

#### Veggie Meter

The Veggie Meter is a non-invasive, portable device that uses reflection spectroscopy to quantify skin carotenoids, a biomarker of F&V intake [33]. Scores can range from 0 to 800, where every 100 points roughly corresponds to one cup of vegetables [34]. The Veggie Meter is a validated measure for assessing F&V intake and is sensitive enough to detect change over time [35, 36]. Skin carotenoids were measured in triplicate (each reading takes 15–20 s) [37].

#### Surveys

Surveys were self-administered online unless participants requested a paper copy or Harvest Share staff assistance. All surveys were available in English, simplified Chinese, and Spanish. Each survey took approximately 45 min to complete, and all CSA and CC participants received a $40 gift card for completing the survey and in-person Veggie Meter assessment at baseline and another $40 gift card for completing these same assessments at follow-up. All survey responses included “don’t know/not sure” and “decline to state” options, which were treated as missing data for the present analysis.

##### Self-reported F&V intake

Participants were asked a series of questions related to F&V intake. First, they reported how many total cups of F&V they ate yesterday. Then, depending on which CSA program or comparison community individuals lived in, they reported the frequency and quantity of intake during the last month for 26 F&V specific to either the Chinese (BG, Chinatown, Flushing) or Mexican (AFF, South Bronx) community. The 26 produce items listed on this measure were selected by the Harvest Share team to include produce that were common within Chinese or Mexican culture—as identified in brief surveys and qualitative interviews conducted during our formative work [26]—; part of the intended CSA offerings, as indicated by our farm partners’ seasonal growing plans; or expected to impact skin carotenoid readings, based on a review of peer-reviewed literature, textbooks, and dietary references (see *Veggie Meter* section). As such, the list of F&V was not intended to be comprehensive and included items that were not provided by the CSA program (e.g., cantaloupes, mangoes) but that were generally available for purchase in the community.

For each of these 26 items, participants indicated if they consumed that item and, if yes, the frequency of consumption (8 response options) and quantity consumed each time (4 response options in cups for larger vegetables; 5 response options in tablespoons for herbs). We created two variables from these responses: (1) total number of different F&V consumed in the last month and (2) total number of cups of F&V consumed per day in the last month. The first variable was created by summing the number of produce items the participant indicated they had eaten last month. If individuals were missing responses to whether they consumed 6 or more of the 26 items, both the total count and cups variable were set to missing. To determine the total number of cups of F&V individuals consumed per day in the last month, we recoded all frequencies to reflect monthly intake and all intake amounts in terms of cups. Frequency was then multiplied by intake to get the total number of cups of produce consumed per day in the last month.

##### Knowledge of F&V

Individuals were asked to indicate which of the F&V from the aforementioned 26-item list they did not know how to prepare or cook. Individuals could select as many produce items as applicable, and the number of selected items was summed to create an overall variable.

##### Ethnic pride and interracial harmony

Ethnic pride and interracial harmony were measured using the Multigroup Ethnic Identity Measure [38]. Individuals indicated on a 4-point Likert scale (1 = strongly disagree to 4 = strongly agree) how much they agreed or disagreed with 5 statements related to ethnic pride (e.g., “I have a lot of pride in my race/ethnicity group(s) and its accomplishments”) and 6 items related to interracial harmony (e.g., “I often spend time with people from racial/ethnic groups other than my own”). Responses were averaged across each subscale, with higher scores indicating greater ethnic pride or interracial harmony (Cronbach’s α = 0.93 and 0.68 for ethnic pride and interracial harmony, respectively). Individuals missing responses to 2 or more items had their overall score set to missing.

##### Perceived stress

Perceived stress was measured using the 10-item Perceived Stress Scale developed by Cohen et al. [39] Individuals indicated on a 5-point Likert scale (0 = never to 4 = very often) how often they had certain feelings and thoughts during the last month (e.g., “In the last month, how often have you felt nervous and ‘stressed’”). The 4 positive items were reversed coded, and responses were summed across the 10 items such that higher scores indicated greater perceived stress (Cronbach’s α = 0.77). Individuals missing responses to 3 or more items had their overall score set to missing.

##### Happiness

Happiness was assessed using two scales to capture both independent [40] and interdependent [41] happiness domains. Independent happiness was assessed using four items where participants indicated on a sliding scale (7-point Likert scale ranging from 0 to 6) the degree to which they felt each statement described them (e.g., “In general, I consider myself…” with response options ranging from 0=“not a very happy person” to 6=“a very happy person”). The 1 positively worded item was reverse coded, and responses across the 4 items were averaged to create an overall score (Cronbach’s α = 0.67). The overall score was set to missing if individuals were missing responses to 2 or more items.

Interdependent happiness was measured using 9 items with a 5-point Likert scale (1 = completely disagree to 5 = completely agree). Individuals indicated how much they agreed or disagreed with these statements (e.g., “I make significant others happy”), and responses were averaged across the 9 items to create an overall score (Cronbach’s α = 0.91). Individuals missing responses to 3 or more items had their overall score set to missing. Higher scores indicate greater happiness on both the independent and interdependent scales.

##### Inclusion of community in self

Inclusion of community in self was assessed using a single item visual representation developed by Mashek et al. [42] Individuals were shown 6 different Venn diagrams with varying degrees of overlap, in which each circle represented either the community or oneself. Individuals were asked to select the picture that best describes their relationship with the community at large. Individuals received a score ranging from 1 to 6, with higher scores representing greater alignment with the community at large.

##### Sense of belonging

Sense of belonging was measured using a single item: “I belong and am accepted in the US”. Individuals indicated how much they agreed or disagreed with the statement using a 4-point Likert scale (1 = strongly agree to 4 = strongly disagree) [43].

##### Socio-demographics 

The following socio-demographics were self-reported at baseline: age, sex, race and ethnicity, nativity, preferred language, income, education level, and household size. Preferred language refers to the language in which the baseline survey was completed.

#### Key informant interviews

At the end of GS1, we invited 8 individuals per language-preference group (*n* = 24 total; English, Chinese, or Spanish; selection occurred via random number generator) to take part in semi-structured interviews. The NYU study team reached out to selected participants via email or phone (based on participant preference). English- and Spanish-speaking participants were referred to the Center for Evaluation and Applied Research (CEAR) at The New York Academy of Medicine (NYAM) to schedule interviews; Mandarin-speaking participants scheduled interviews with one of the bilingual Harvest Share staff members who were trained on interview protocols by NYAM staff. Only one English-speaking and one Spanish-speaking individual did not participate after expressing interest; a new English-speaking participant was invited after scheduling difficulties. A total of *n* = 23 participants participated, of which *n* = 16 participated in the BG CSA (*n* = 11 completed in English; *n* = 5 completed in Mandarin) and *n* = 7 (all Spanish-speaking) participated in the AFF CSA. Interviews lasted up to 60 min and covered their experience with and perspectives on the Harvest Share CSA and other program activities, as well as recommendations for improvement. Interviews were conducted between March and May 2024 over the phone and in participants’ preferred language. Participants provided verbal consent at the start of each interview and received a $50 gift card via email, text, or mail, depending on preference.

### Analysis

#### Quantitative analysis

The present analysis is limited to individuals who completed both the survey and Veggie Meter at baseline and were thus fully enrolled in the program (*n* = 542; excluded *n* = 1 who only completed the survey). Retention at follow-up is similarly based on the number of individuals who completed *both* the survey and Veggie Meter at that time (excluded *n* = 22 who only completed one component). Descriptive statistics for socio-demographics and primary and secondary outcomes at baseline were examined for the sample overall and stratified by Harvest Share CSA and comparison community participants. Descriptive statistics for socio-demographics were examined further stratified by evaluation time points.

A mixed effect linear regression model was used to examine the impact of the Harvest Share program on the primary and secondary outcomes. Unadjusted and adjusted models were explored, with separate models for each outcome of interest. In all models, intervention group was a binary variable corresponding to participation in the Harvest Share CSA or comparison community assessments. Time was treated as a binary variable (0 = pre, 1 = post), and the effect of Harvest Share on our outcomes was based on the regression coefficient for the intervention group x time interaction term. Adjusted models included the following covariates as fixed effects to account for differences between groups: age; sex; race and ethnicity; nativity; preferred language; income; education level; and household size. To account for the repeated measures among individuals, study ID, nested within neighborhood, was included as a random effect. All data cleaning and analyses were conducted in SAS version 9.4.

#### Qualitative analysis

Interviews were transcribed and translated, as needed. The coding scheme reflected the evaluation objectives and included pre-identified topics, as well as topics deriving from the data themselves. Transcripts were analyzed using descriptive, thematic analysis consistent with a content analysis approach and focused on findings most relevant to program evaluation and improvement [44]. This iterative process continued until the analytical team reached consensus regarding central themes. Each transcript was coded by two trained coders, and coding discrepancies were discussed and resolved through consensus. All data were maintained and analyzed in NVivo.

## Results

### Quantitative findings

An overview of socio-demographics characteristics of Harvest Share participants (*n* = 542) at GS1 baseline is provided in Table 1, overall and by CSA vs. comparison community participants. Supplementary Table 1 shows this data by specific CSA program and comparison community. A total of *n* = 153 CSA participants (*n* = 109 BG, *n* = 44 AFF) and *n* = 389 comparison community members (*n* = 91 Chinatown, *n* = 115 Flushing, *n* = 183 South Bronx) completed the baseline evaluation. The average produce pickup rate was 88% (91% for BG, 81% for AFF; data not shown), with 12% of pick-ups being done by alternate contacts (10% for BG, 25% for AFF; data not shown). At GS1 follow-up, 84% of participants were still engaged with the CSA (88% BG, 73% AFF). Retention rates for the comparison communities were similar: 83% overall (87% Chinatown, 87% Flushing, 78% South Bronx).

**Table 1:** Descriptive statistics for Harvest Share participants overall and by intervention group for Growing Season 1 baseline

	Overall (*n* = 542)	Intervention group	
		CSA Participants (*n* = 153)	Comparison Communities (*n* = 389)
Age (years), mean (SD)	43.9 (15.1)	41.9 (13.2)	44.7 (15.7)
Gender, *n* (%)			
Man	96 (17.7)	32 (20.9)	64 (16.5)
Woman	435 (80.4)	115 (75.2)	320 (82.5)
Other	10 (18.5)	6 (3.9)	4 (1.0)
Missing	1	0	1
Nativity, *n* (%)			
Born in US	128 (24.2)	75 (49.7)	53 (14.0)
Born outside of US	401 (75.8)	76 (50.3)	325 (86.0)
Missing	13	2	11
Race and ethnicity, *n* (%)			
Asian	258 (48.4)	57 (38.3)	201 (52.3)
Hispanic/Latino	196 (36.8)	40 (26.8)	156 (40.6)
Non-Hispanic White	39 (7.3)	39 (26.2)	0 (0.0)
Multiracial	34 (6.4)	12 (8.1)	22 (5.7)
Another race or ethnicity	6 (1.1)	1 (0.7)	5 (1.3)
Missing	9	4	5
Language preference, *n* (%)			
Chinese	170 (31.4)	18 (11.8)	152 (39.1)
English	160 (29.5)	101 (66.0)	59 (15.2)
Spanish	212 (39.1)	34 (22.2)	178 (45.8)
Education, *n* (%)			
< High school	117 (23.0)	24 (16.1)	93 (25.8)
Some high school	103 (20.2)	12 (8.1)	91 (25.3)
High school or equivalent	162 (31.8)	29 (19.5)	133 (36.9)
≥College	127 (25.0)	84 (56.4)	43 (11.9)
Missing	33	4	29
Annual household income, *n* (%)			
<$12,000	129 (23.8)	22 (14.4)	107 (27.5)
$12,000-$34,999	120 (22.1)	24 (15.7)	96 (24.7)
$35,000-$49,999	47 (8.7)	16 (10.5)	31 (8.0)
$50,000-$99,999	59 (10.9)	30 (19.6)	29 (7.5)
≥$100,000	53 (9.8)	39 (25.5)	14 (3.6)
Unknown/missing	134 (24.7)	22 (14.4)	112 (28.8)
Household size, *n* (%)			
1 person	52 (9.7)	26 (17.2)	26 (6.8)
2 persons	116 (21.7)	49 (32.5)	67 (17.5)
≥ 3 persons	367 (68.6)	76 (50.3)	291 (75.8)
Missing	7	2	5

“Another race or ethnicity” includes the following self-selected groups: American Indian/Alaskan Native, Non-Hispanic Black, Southwest Asian and North African, Native Hawaiian and Pacific Islander

High school or equivalent includes high school, Associate’s degree, technical school, and vocational school

*CSA* Community Supported Agriculture, *US* United States

As shown in Table 1, participants were, on average, in their early 40s (44 years, SD: 15); self-identified as female (80%) and Asian (48%) or Hispanic/Latino (37%); and from households with ≥ 3 individuals (69%). A higher proportion of comparison community participants were born outside of the US (86% vs. 50%, respectively) and preferred a language other than English (85% vs. 34%, respectively) compared to CSA participants. CSA participants generally had a higher education level than comparison community participants, and annual household income skewed slightly lower for comparison community participants. Still, 32% of CSA participants paid for their shares weekly via SNAP (26% BG, 64% AFF; data not shown).

Tables 2 and 3 present results from the unadjusted and adjusted linear mixed effects models, respectively. We focus on findings from the adjusted models as significance did not differ across approaches and these estimates more fully account for confounding. There were significant differences in the mean change over time between the CSA and comparison community groups for our primary outcome (Veggie Meter, *p* = 0.02) and two diet-related outcomes (F&V consumed in the last month, *p* < 0.0001; F&V knowledge, *p* = 0.0004).

**Table 2:** Mean difference in primary and secondary outcomes among Harvest Share CSA and comparison community participants from unadjusted mixed effect linear regression models

	Unadjusted mean difference		
	Harvest Share	Comparison Community	*p*-value for group x time interaction
Primary Outcome			
Veggie Meter score	-9.61 (-22.81, 3.58)	**-25.68 (-33.88**, -17.49)*	**0.04**
Secondary Outcomes			
Dietary outcomes			
Total cups of F&V yesterday, 1-item	0.20 (-0.05, 0.46)	-0.05 (-0.21, 0.12)	0.11
Total cups of F&V last month, 26-item	-0.10 (-0.75, 0.56)	-0.35 (-0.77, 0.07)	0.52
# of F&V last month, 26-item	**3.46 (2.69**, 4.22)*	0.02 (-0.46, 0.50)	**< 0.0001**
# of F&V don’t know how to prepare, 26-item	**-1.55 (-2.33**, -0.78)*	-0.13 (-0.62, 0.37)	**0.002**
Social outcomes			
Ethnic pride	-0.13 (-0.31, 0.04)	0.06 (-0.04, 0.17)	0.06
Interracial harmony	0.05 (-0.05, 0.15)	-0.01 (-0.08, 0.07)	0.37
Perceived stress	-0.62 (-1.56, 0.33)	0.24 (-0.39, 0.88)	0.14
Happiness - Independent	0.12 (-0.05, 0.29)	0.05 (-0.06, 0.15)	0.45
Happiness - Interdependent	0.02 (-0.14, 0.18)	0.03 (-0.07, 0.14)	0.90
Inclusion of community in self	-0.12 (-0.38, 0.13)	0.05 (-0.12, 0.22)	0.25
Sense of belonging	-0.07 (-0.22, 0.08)	-0.09 (-0.18, 0.00)	0.83

Mean differences are from separate mixed effect linear regression models for each outcome. Study ID (to account for repeated measures among participants) nested within neighborhood (to account for clustering of individuals within neighborhoods) were random effects, and time (pre/post), group (CSA/Comparison Community), and time x group were included as fixed effects

*F&V* Fruits and vegetables

Bold values and * indicate statistical significance, *p*<0.05

**Table 3:** Mean difference in primary and secondary outcomes among Harvest Share CSA and comparison community participants from fully adjusted mixed effect linear regression models

	Adjusted mean difference		
	Harvest Share	Comparison Community	*p*-value for group x time interaction
Primary Outcome			
Veggie Meter score	-7.43 (-21.30, 6.44)	**-27.29 (-36.27**, -18.30)*	**0.02**
Secondary Outcomes			
Dietary outcomes			
Total cups of F&V yesterday, 1-item	0.18 (-0.07, 0.44)	-0.11 (-0.28, 0.06)	0.06
Total cups of F&V last month, 26-item	-0.02 (-0.69, 0.64)	-0.45 (-0.89, 0.00)	0.30
# of F&V last month, 26-item	**3.74 (2.94**, 4.53)*	0.05 (-0.48, 0.57)	**< 0.0001**
# of F&V don’t know how to prepare, 26-item	**-1.60 (-2.28**, -0.93)*	-0.13 (-0.57, 0.32)	**0.0004**
Social outcomes			
Ethnic pride	-0.11 (-0.29, 0.07)	0.08 (-0.04, 0.20)	0.08
Interracial harmony	0.06 (-0.05, 0.17)	-0.0056 (-0.08, 0.07)	0.32
Perceived stress	-0.71 (-1.67, 0.26)	0.28 (-0.39, 0.95)	0.10
Happiness - Independent	0.12 (-0.05, 0.28)	0.06 (-0.05, 0.17)	0.59
Happiness - Interdependent	0.05 (-0.12, 0.21)	-0.01 (-0.13, 0.10)	0.56
Inclusion of community in self	-0.11 (-0.36, 0.14)	-0.01 (-0.19, 0.16)	0.54
Sense of belonging	-0.08 (-0.23, 0.07)	-0.05 (-0.14, 0.05)	0.70

Adjusted mean differences are from separate mixed effect linear regression models for each outcome. Study ID (to account for repeated measures among participants) nested within neighborhood (to account for clustering of individuals within neighborhoods) were random effects, and the following variables (in addition to time [pre/post], group [CSA/Comparison Community], and time x group) were included as fixed effects to account for differences between groups: age, gender, race and ethnicity, nativity, preferred language, income, education level, and household size

*F&V *Fruits and vegetables

Bold values and * indicate statistical significance, *p*<0.05

**Fig. 1:**
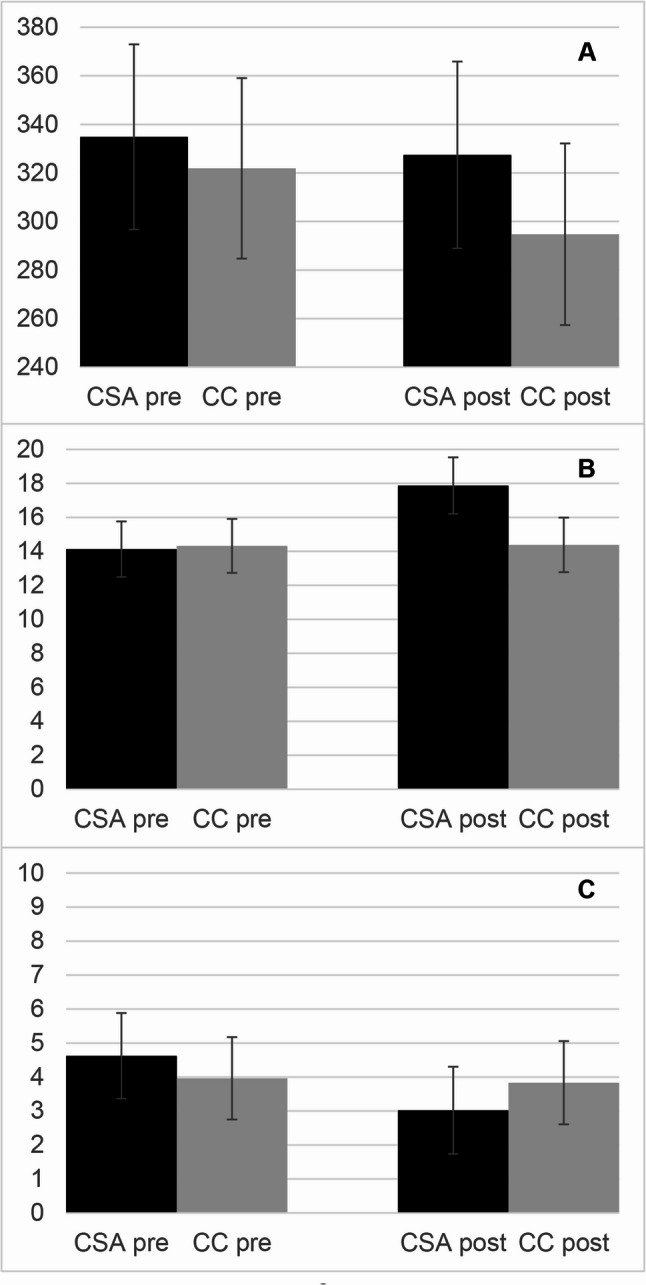
Impact of Harvest Share on Veggie Meter scores (Panel **A**), number of different fruits and vegetables consumed (Panel **B**), and number of fruits and vegetables don’t know how to prepare (Panel **C**) for community supported agriculture (CSA) vs. comparison community (CC) participants during Growing Season 1

The adjusted mean difference in Veggie Meter scores from baseline to follow-up was −7.43 (95%CI: −21.30, 6.44) for the CSA group and −27.29 (95%CI: −36.27, −18.30) for comparison community participants. For the dietary outcomes, CSA participants reported a significant increase in the number of different types of F&V they consumed in the last month (26-item measure; 3.74 [95%CI: 2.94, 4.53]) as well as in their knowledge of F&V preparation (mean difference for *not* knowing how to prepare F&V: −1.60 [95%CI: −2.28, −0.93]). Comparison community participants did not report significant changes in either of these outcomes (0.05 [95%CI: −0.48, 0.57] and −0.13 [95%CI; −0.57, 0.32], respectively). There were no significant findings for the other secondary outcomes examined. Supplementary Tables 3–5 provide the full model specifications for significant findings. Figure 1 visually depicts the significant results from Table 3.

### Qualitative findings

Table 4 provides an overview of the interviewees' sociodemographic characteristics. Participant feedback on program implementation details and recommendations for the future is provided in Supplementary Table 6. Findings that relate to perceived program impact are detailed below with relevant quotes in Table 5. Income levels indicated below are defined as: low (<$25,000), medium ($25,000-$99,999), and high (≥$100,000).

**Table 4:** Descriptive statistics for participants in Growing Season 1 interviews

	Overall (*n* = 23)
Age (years), mean (SD)	46.7 (13.5)
Gender, *n* (%)	
Man	4 (17.4)
Woman	19 (82.6)
Nativity, *n* (%)	
Born in US	9 (39.1)
Born outside of US	14 (60.9)
Race and ethnicity, *n* (%)	
Asian	10 (43.5)
Hispanic/Latino	7 (30.4)
Non-Hispanic White	5 (21.7)
Multiracial	1 (4.3)
Language preference, *n* (%)	
Chinese	5 (21.7)
English	11 (47.8)
Spanish	7 (30.4)
Education, *n* (%)	
<High school	4 (17.4)
Some high school	2 (8.7)
High school or equivalent	7 (30.4)
≥College	10 (43.5)
Annual household income, *n* (%)	
<$12,000	8 (34.8)
$12,000-$34,999	3 (13.0)
$35,000-$49,999	1 (4.3)
$50,000-$99,999	4 (17.4)
≥$100,000	7 (30.4)
Household size, *n* (%)	
1 person	2 (8.7)
2 persons	7 (30.4)
≥3 persons	14 (60.9)

Low-income considered <$35,000 per year; High-income considered ≥$100,000 per year

1 individual’s partner completed the interview instead of the enrolled participant

*US* United States

**Table 5:** Relevant quotes from interview participants highlighting the qualitative themes

Theme	Sample quotes
Food security and finances	*“Everything is so expensive. Sometimes*,* when we go buy groceries*,* we can’t buy the same amounts that we’re used to buying.”* (Spanish-speaking, low income) *“The medication my husband was taking was very expensive because it’s heart medication. So*,* we had to figure out how to pay for it. So*,* this help that [the program] provided was wonderful because we were able to get the money to pay for the medication.”* (Spanish-speaking, low income)
Communal meals and produce sharing	*“I would be like*,* ‘Hey*,* we’ve got this and this and this.’ Then [my in-laws]*,* they’re like*,* ‘Oh*,* that’s really good. We’d like some of that’ […] Definitely*,* I think we eat more together.”* (English-speaking, Chinese-identifying, high income)
Ethnic pride and cross-cultural learning	*“I was very interested in this [program] because other places give out food*,* but they’re not Mexican produce…I do think it’s important for [my children] to also consume Mexican produce and produce from here too because they have two cultures. So*,* we don’t want our roots to be lost on them because we fail to teach them to consume Mexican produce.”* (Spanish-speaking, low income) *“I feel more comfort in being able to figure out what to do with some things and just discovering other ways to use the Chinese vegetables that in the past I might’ve been like—I wouldn’t buy that because I don’t know what it is*,* and I don’t know what to do with it.”* (English-speaking, high income)
Sense of community and interracial harmony	*“I personally don’t speak much English. We speak Spanish and they speak [Chinese]*,* but we managed to communicate either way. It was a nice thing because we would find a way to communicate. And we met different people there. I have even bumped into people that we met there*,* and we say ‘Hi*,* how are you?’ So*,* we’ve made new friends.”* (Spanish-speaking, low income) *“For me it was like a neighborhood apart*,* because I’m not far from there. So*,* for me*,* like it was like another place…But now I consider it to be mine*,* because I’m going there.”* (Spanish-speaking, low income)
Relationship with green spaces and gardening at home	*“It brings back beautiful memories because I was growing that in Mexico. We had farmland and we always grew up growing vegetables…We felt connected to our childhood that we had*,* because we grew all this there with my parents.”* (Spanish-speaking, low income)
Mental health	*“I mean*,* it made me happier being able to see green*,* escape the concrete*,* just everything*,* and be able to go see these plants*,* even touch these plants. It was awesome.”* (English-speaking, high income) *“For me*,* it’s less stress*,* because you go—and you don’t even know what they’re going to give you—and when you arrive and you see the box […] The stress that it takes away from you is that you don’t have to say*,* ‘Oh*,* I have to buy this or I have to buy that.’”* (Spanish-speaking, low income)
Health behaviors	*“It was fun for us to get there because it was also a form of exercise for us. We walked there and everything. I mean*,* there were no issues. It’s easy and fun for the children.”* (Spanish-speaking, low income) *“I definitely developed healthier food habits ‘cause you go every week*,* and then eventually it becomes a habit that you need vegetables in your refrigerator or at home. Just because you’ve always had vegetables*,* at some point it becomes like something’s missing if you don’t get it. I think that really helped out.”* (English-speaking, Chinese-identifying, high income) *“I think it just adds to [my mother’s] overall well-being; to eat more vegetables*,* get more fiber*,* and it just helps because she is interested in it. And she talks about it more*,* so it’s just part of her person now. Like here’s what I eat daily*,* and prepare for myself*,* and here’s the vegetables we get every week. And then she takes a walk in the park or meet up with friends. So*,* overall*,* it has uplifted her daily life.”* (English-speaking, Chinese-identifying, middle income)

#### Food security and finances

Most respondents indicated that the subsidized produce program helped them and their family save on costs. Some individuals indicated that, without the program, they wouldn’t have been able to afford as many vegetables. Participants described using the money saved to purchase additional food items, pay bills, and purchase other needed items (e.g., medications, school supplies). Some solely reported saving money due to the program and not redirecting it in other ways.

#### Communal meals and produce sharing. 

Most participants reported that they shared produce and meals they made with the CSA produce with family and friends, as well as with new individuals. Reasons for increased food sharing included: excitement about the fresh produce; not wanting to waste produce; and knowing someone who would be interested in using the vegetables. Some interviewees noticed an increase in the number of meals they shared and discussions they had with others about food and meal preparation throughout the program. A few interviewees shared that they or their families started to cook more often, and some described how their children learned about vegetables and became more involved in the cooking process.

#### Ethnic pride and cross-cultural learning 

Participants frequently reported that the CSA produce offerings helped them and their families feel more connected to their culture. This sentiment was shared by participants identifying as either Mexican or Chinese. Participants who did not identify as Chinese or Mexican reported that participating in the program taught them how to use produce from other cultures.

#### Sense of community and interracial harmony

More than half of interviewees reported that the program positively impacted their own or their family’s sense of community by allowing them to interact with other community members whom they may not have otherwise had the chance to meet, including individuals from other racial and ethnic groups. Participants also reported that they now felt BG was a part of their community and a space where they were welcomed.

#### Relationship with green spaces and gardening at home 

Generally, interviewees enjoyed the weekly pick-ups from the BG farm as they allowed them to be surrounded by nature, enjoy fresh air, take in the rooftop views, and escape from the city. Participants also noted that being on the farm helped them feel more connected to their childhood. Approximately half noted that the experience made them want to spend more time outside or in nature or grow things at home.

#### Mental health

Most participants reported that visiting the farm made them and their families feel happier, more relaxed, or in an overall better mood. They attributed this to the farm’s peaceful environment, scenic views, and abundance of plants and crops they could admire during their visits. A few interviewees reported the CSA reduced their stress levels as they no longer had to spend as much time thinking or worrying about what type of produce to buy and what meals to prepare each week.

#### Health behaviors 

Participants shared that the program helped them start incorporating healthier habits (e.g., consuming more vegetables, walking more) into their daily lives. Most interviewees indicated that walking to the farm to pick up their produce each week increased the amount of physical activity they engaged in weekly. Participants also reported consuming more vegetables, either because they did not want the fresh vegetables to go to waste or because vegetable consumption became part of their routine. Participants also noticed improvements to their health and well-being due to these lifestyle changes.

## Discussion

In our mixed methods examination of year-one data from Harvest Share, participation in a tailored, cost-offset CSA positively impacted participants’ quantity and variety of F&V intake and F&V preparation knowledge. While quantitative findings for ethnic pride, interracial harmony, and perceived stress were null, qualitative interviews captured beneficial changes in these measures. Overall, our findings mirror those from our pilot study (*n* = 38) where CSA participation significantly improved Veggie Meter scores, number of F&V consumed, and F&V preparation knowledge [24]. This is the first study, to our knowledge, to examine the impact of a full-scale, cost-offset CSA tailored to local community preferences that included a large number of participants that are individuals of color.

In the present study, we found that CSA participation had a positive impact on F&V intake, similar to previous studies [11, 14, 18]. However, in contrast with previous programs, CSA participation did not promote increased intake; intake non-significantly declined. A few factors may explain why a downward trend in F&V intake was observed among both the CSA and comparison community participants. National data indicates there was decreased vegetable availability (-2.2% from 2022) and elevated vegetable prices (from May to September relative to 2022) across the US during 2023, which likely impacted produce availability and pricing in NYC during the months of our study [45]. Additionally, 2023 was also the hottest year on record (recently surpassed by 2024) [46], which may have made commuting to stores for fresh produce uncomfortable, resulting in decreased intake. While we cannot ascertain the exact reasons for the lower Veggie Meter scores in both groups at the end of follow-up, CSA participation was protective of the “expected” decline seen in the comparison communities. These findings also highlight the importance of including comparison groups in the design of CSA studies to capture “usual” trends that may be impacting findings. Notably, many previous studies of cost-offset CSAs have had no comparison groups [14, 15, 19, 20, 22], of which two of these showed no change in vegetable intake [19, 20]. Without a comparison group, we may have concluded that the CSA had no impact (or a negative impact) on F&V intake, rather than serving as protection against expected declines in intake.

In addition to observing a positive impact of the CSA program on F&V intake as measured by the Veggie Meter, we also observed a positive association between CSA participation and two self-reported diet and dietary behaviors: variety of F&V consumed during the last month and F&V preparation knowledge. The 2020–2025 Dietary Guidelines for Americans centers the importance of vegetable variety for overall health and have set specific targets for vegetable types, such as 1.5 cups/week of dark green vegetables and 5.5 cups/week of red and orange vegetables [1]. Thus, improving participants’ vegetable variety—with or without associated increases in quantity—and the associated knowledge to prepare those vegetables, as in Harvest Share, are promising means of improving overall diet quality and reducing risk for related adverse health outcomes [1, 4, 47].

The association between CSA participation and increased variety of vegetables in the diet in the present study is consistent with findings from cost-offset [12, 15, 22] and standard [48–50] CSA programs [10], and has also been observed in the context of no increases in the quantity of vegetable intake [19, 20]. Previous reports have attributed this increase in variety to participants relying on the seasonally available vegetables (including those which they may be unfamiliar with) provided via the CSA as their primary vegetable source [10, 20]. This appeared to be true in Harvest Share, with interviewees indicating that they had to learn how to incorporate less familiar vegetables into their existing diets using our programming and recipe cards—as well as self-teaching—to make the most of their CSA share. This increase in knowledge and approach to learning how to navigate less familiar vegetables is similar to reports from previous studies [10, 12, 20, 23].

While we did not observe preliminary impacts of the CSA program on secondary quantitative outcomes related to social and mental health, our qualitative analysis showed the program had positive impacts on constructs of interest: ethnic pride, interracial harmony, perceived stress, happiness, and community/social connection. It is possible that the null findings for some of these results are due to the lower internal consistency of some of the scales (i.e., Cronbach’s α ≥ 0.70 is generally the threshold for “good” reliability [51]). These findings also highlight the importance of using mixed-methods approaches to understand subjective experiences in multicultural contexts [52]. Qualitative research provides participants with an opportunity to share about their experiences in a more personal, unstructured format relative to quantitative approaches [53]. While the survey items we selected have been previously validated for use in multiple research contexts, the interview prompts were tailored to the Harvest Share programming and thus may have prompted more meaningful recollections that allowed us to see the impacts of the CSA program. Previous CSA studies have not been able to examine the potential impact of CSAs on ethnic pride nor interracial harmony due to the enrollment of predominantly non-Hispanic White women [10]. However, interviewees’ experiences with Harvest Share’s unique produce tailoring and our focus on bringing together the residents of Sunset Park into one shared space inclusive of all identities indicates that CSAs can foster cross-cultural learning and understanding.

Related to stress and happiness, interviewees generally commented that attending produce pick-ups lifted their moods due to exposure to outdoor green space—something that is often lacking in NYC neighborhoods—, positive social interactions with other community members (as well as program staff), and a sense of stress relief that stemmed from affordable access to fresh vegetables that also “decided for them” the meals that they would be preparing for the week. For some participants, engaging with the CSA (either during pick-up or while cooking) improved their moods, as the program reminded them of the produce that they grew or ate with their family when they were young.

A key finding from the qualitative research is that participants felt improved social connection. Participants underscored that even when interactions with other participants or staff were brief, they appreciated the sense of community it created, similar to reports from other CSA programs [20, 54]. This increased sense of belonging and community connectedness have been identified as facilitators of CSA engagement [16, 17], and thus by creating these opportunities for positive social connection, we aim to indirectly improve F&V intake and overall health [55, 56]. To additionally explore these themes, we have conducted a ripple effect mapping evaluation, with results to be reported at a later date.

Relatedly, many participants reported that engagement with the CSA improved their physical (in addition to mental) health, in line with findings from previous studies [10, 12, 23, 54]. By engaging with the CSA, participants reported engaging in healthier behaviors including increasing vegetable intake, time spent walking, the number of diet and health-related conversations, and cooking more meals at home. As in other studies [23, 55], participants reported increased opportunities to share recipes, produce, or cooked meals with friends, family members, and coworkers.

This study has numerous strengths. Importantly, through our multisector collaboration, we addressed a community need for affordable, familiar produce in the neighborhood. We were able to reach populations across multiple socioeconomic and identity groups, and participants felt welcomed into Harvest Share through our programming. Importantly, we saw high retention rates during GS1, even in the comparison communities where CSA programming was not offered. This speaks to the importance of partnering with trusted community-based organizations to facilitate recruitment and provide safe physical spaces to conduct the evaluations; the ability of the bilingual Harvest Share staff to instill trust in community members; and the importance of aligning incentive amounts with the study burden. Further, among CSA participants, we saw high pick-up rates, similar to those reported in a recent multicenter randomized controlled trial (average of 88% in GS1 vs. median of 88% in previous research [57]), which highlights our responsiveness to community needs for fresh, local produce [9, 24, 26] and implementation of key facilitators to CSA engagement and retention [17]. Lastly, our study incorporated an objective measure of F&V intake (the Veggie Meter), which addresses recall bias known to impact dietary intake assessed via self-report [58]. The Veggie Meter has moderate to high inter- and intradevice repeatability [59] and is sensitive enough to detect longitudinal change in scores [35, 36], making it a notable strength of dietary assessment in Harvest Share. Outside of our pilot study [24], only one other CSA examination has used skin carotenoids to determine F&V intake [60]. Notably, the Veggie Meter had high community acceptability and seemed to promote retention.

Regarding limitations, while Harvest Share’s study design is robust in its inclusion of comparison communities, we did not use probabilistic sampling methods and thus cannot assume that the comparison community participants are necessarily representative of the larger communities’ populations. Because we did not examine an extensive list of F&V individuals consumed, it is possible that individuals did not increase *overall* vegetable variety and instead just increased variety amongst the list of 26 items assessed. There are also some limitations related to the Veggie Meter data collection. Due to the nature of the data collection methods, Veggie Meter readings occurred outdoors, which can affect the observed readings [37]. Despite the strengths of the Veggie Metter, recent literature also suggests that the machine’s performance may vary across participant-level factors, with lower intradevice repeatability among Black individuals and individuals with lower scores [61, 62], and that performance can differ across devices [59, 63]. Harvest Share uses four different Veggie Meter devices across the communities; however, we did not systematically record which machine was used in which community at each time point during GS1. As such, the present analysis does not adjust for Veggie Meter machine; efforts are underway to pull this data directly from the machines and map to participants for future Harvest Share analyses. Finally, while we aimed to capture changes in social secondary outcomes via our surveys, the selected measures may not be sensitive to change for our populations of interest *or* a longer time period is needed to observe changes in these quantitative measures. We will continue exploring these measures in a future primary outcomes paper.

## Conclusions

At the end of GS1, we found that participation in a cost-offset CSA tailored to local community preferences positively impacted the quantity and variety of fruits and vegetables consumed as well as knowledge of F&V preparation in a population that has historically not participated in CSAs. Interviews with participants highlighted the benefits of the program on ethnic pride, interracial harmony, perceived stress, happiness, and social connection/sense of belonging. The impacts of Harvest Share on our primary and secondary outcomes across all four planned growing seasons will be examined in a future analysis. 

## Supplementary Information

Supplementary Material 1.

## Data Availability

The datasets generated and/or analyzed during the current study are not publicly available due to ongoing data collection efforts and participant privacy concerns but are available from the corresponding author on reasonable request.

## Abbreviations

AFF: Angel Family Farm
BG: Brooklyn Grange
CC: Comparison Community
CEAR: Center for Evaluation and Applied Research
CI: Confidence Interval
CPC: Chinese-American Planning Council
CSA: Community-Supported Agriculture
EBT: Electronic Benefit Transfer
F&V: Fruits & Vegetables
IRB: Institutional Review Board
NYAM: The New York Academy of Medicine
NYC: New York City
NYU: New York University
P.S.: Public School
SD: Standard Deviation
SNAP: Supplemental Nutrition Assistance Program
US: United States
WIC: Women, Infants, and Children
