# Supplementary material for: Preliminary trial findings from Harvest Share: a cost-offset community supported agriculture model to improve diet in New York City
Source: Int J Behav Nutr Phys Act. 2026 Jul 11;23:73. doi: 10.1186/s12966-026-01906-z (PMC13393813; doi:10.1186/s12966-026-01906-z)
Supplement: Supplementary file 1 — Supplementary Material 1. [file 12966_2026_1906_MOESM1_ESM.docx]

**Supplementary Table 1.** Descriptive statistics for Harvest Share participants by CSA and comparison community subgroups for Growing Season 1 baseline

|  | **CSA** | |  | **Comparison community** | | |
| --- | --- | --- | --- | --- | --- | --- |
|  | **Brooklyn Grange (*n*=109)** | **Angel Family Farm (*n*=44)** |  | **Chinatown**  **(*n*=91)** | **Flushing**  **(*n*=115)** | **South Bronx**  **(*n*=183)** |
| **Age (years), mean (SD)** | 40.1 (13.3) | 46.3 (12.0) |  | 44.2 (14.0) | 51.9 (18.4) | 40.4 (12.8) |
| **Gender, *n* (%)** |  |  |  |  |  |  |
| Man | 29 (26.6) | 3 (6.8) |  | 19 (20.9) | 22 (19.3) | 23 (12.6) |
| Woman | 74 (67.9) | 41 (93.2) |  | 72 (79.1) | 89 (78.1) | 159 (86.9) |
| Other | 6 (5.5) | 0 (0.0) |  | 0 (0.0) | 3 (2.6) | 1 (0.5) |
| *Missing* | *0* | *0* |  | *0* | *1* | *0* |
| **Nativity, *n* (%)** |  |  |  |  |  |  |
| Born in US | 63 (58.3) | 12 (27.9) |  | 25 (27.8) | 14 (12.8) | 14 (7.8) |
| Born outside of US | 45 (41.7) | 31 (72.1) |  | 65 (72.2) | 95 (87.2) | 165 (92.2) |
| *Missing* | *1* | *1* |  | *1* | *6* | *4* |
| **Race and ethnicity, *n* (%)** |  |  |  |  |  |  |
| Asian | 57 (52.8) | 0 (0.0) |  | 87 (95.6) | 114 (99.1) | 0 (0.0) |
| Hispanic/Latino | 5 (4.6) | 35 (85.4) |  | 3 (3.3) | 1 (0.9) | 152 (85.4) |
| Non-Hispanic White | 35 (32.4) | 4 (9.8) |  | 0 (0.0) | 0 (0.0) | 0 (0.0) |
| Multiracial | 10 (9.3) | 2 (4.9) |  | 1 (1.1) | 0 (0.0) | 21 (11.8) |
| Another race or ethnicity | 1 (0.9) | 0 (0.0) |  | 0 (0.0) | 0 (0.0) | 5 (2.8) |
| *Missing* | *1* | *3* |  | *0* | *0* | *5* |
| **Language preference, *n* (%)** |  |  |  |  |  |  |
| Chinese | 18 (16.5) | 0 (0.0) |  | 55 (60.4) | 97 (84.3) | 0 (0.0) |
| English | 89 (81.7) | 12 (27.3) |  | 36 (39.6) | 18 (15.7) | 5 (2.7) |
| Spanish | 2 (1.8) | 32 (72.7) |  | 0 (0.0) | 0 (0.0) | 178 (97.3) |
| **Education, *n* (%)** |  |  |  |  |  |  |
| <High school | 10 (9.3) | 14 (33.3) |  | 19 (23.8) | 15 (14.0) | 59 (34.1) |
| Some high school | 6 (5.6) | 6 (14.3) |  | 19 (23.8) | 30 (28.0) | 42 (24.3) |
| High school or equivalent | 15 (14.0) | 14 (33.3) |  | 22 (27.5) | 46 (43.0) | 65 (37.6) |
| ≥College | 76 (71.0) | 8 (19.0) |  | 20 (25.0) | 16 (15.0) | 7 (4.0) |
| *Missing* | *2* | *2* |  | *11* | *8* | *10* |
| **Annual household income, *n* (%)** |  |  |  |  |  |  |
| <$12,000 | 9 (8.3) | 13 (29.5) |  | 13 (14.3) | 27 (23.5) | 67 (36.6) |
| $12,000-$34,999 | 15 (13.8) | 9 (20.5) |  | 32 (35.2) | 38 (33.0) | 26 (14.2) |
| $35,000-$49,999 | 11 (10.1) | 5 (11.4) |  | 11 (12.1) | 14 (12.2) | 6 (3.3) |
| $50,000-$99,999 | 28 (25.7) | 2 (4.5) |  | 15 (16.5) | 12 (10.4) | 2 (1.1) |
| ≥$100,000 | 35 (32.1) | 4 (9.1) |  | 6 (6.6) | 8 (7.0) | 0 (0.0) |
| Unknown/missing | 11 (10.1) | 11 (25.0) |  | 14 (15.4) | 16 (13.9) | 82 (44.8) |
| **Household size, *n* (%)** |  |  |  |  |  |  |
| 1 person | 23 (21.5) | 3 (6.8) |  | 6 (6.7) | 17 (14.9) | 3 (1.7) |
| 2 persons | 45 (42.1) | 4 (9.1) |  | 17 (19.1) | 29 (25.4) | 21 (11.6) |
| ≥3 persons | 39 (26.4) | 37 (84.1) |  | 66 (74.2) | 68 (59.6) | 157 (86.7) |
| *Missing* | *2* | *0* |  | *2* | *1* | *2* |

CSA, Community Supported Agriculture; US, United States

 “Another race or ethnicity” includes the following self-select groups: American Indian/Alaskan Native, Non-Hispanic Black, Southwest Asian and North African, Native Hawaiian and Pacific Islander

High school or equivalent includes high school, Associate’s degree, technical school, and vocational school

**Supplementary Table 2.** Descriptive statistics (mean (SD)) for Harvest Share participants overall and by intervention group for Growing Season 1 baseline

|  | **Overall (*n*=542)** |  | **Intervention group** | |
| --- | --- | --- | --- | --- |
|  |  |  | **CSA Participants (*n*=153)** | **Comparison Communities (*n*=389)** |
| **PRIMARY OUTCOME** |  |  |  |  |
| Veggie Meter score (range: 0-800) | 330.3 (116.5) |  | 323.8 (103.6) | 332.7 (121.1) |
| *Missing, n* | *12* |  | *8* | *4* |
| **SECONDARY OUTCOMES** |  |  |  |  |
| ***Dietary outcomes*** |  |  |  |  |
| Total cups of F&V yesterday, 1-item | 1.8 (1.3) |  | 1.9 (1.3) | 1.7 (1.3) |
| *Missing, n* | *4* |  | *0* | *4* |
| Total cups of F&V last month, 26-item | 3.7 (5.2) |  | 2.2 (2.0) | 4.2 (6.0) |
| *Missing, n* | *40* |  | *11* | *29* |
| # of different F&V last month, 26-item | 17.1 (5.6) |  | 15.3 (4.9) | 17.8 (5.7) |
| *Missing, n* | *27* |  | *9* | *18* |
| # of F&V don’t know how to prepare, 26-item | 3.2 (4.0) |  | 3.9 (3.9) | 2.9 (4.0) |
| *Missing, n* | *45* |  | *11* | *34* |
| ***Social outcomes*** |  |  |  |  |
| Ethnic pride (range: 1-4) | 3.4 (0.8) |  | 3.3 (0.7) | 3.4 (0.8) |
| *Missing, n* | *96* |  | *32* | *64* |
| Interracial harmony (range: 1-4) | 3.3 (0.6) |  | 3.6 (0.5) | 3.2 (0.5) |
| *Missing, n* | *124* |  | *23* | *101* |
| Perceived stress (range: 0-40) | 16.4 (6.2) |  | 17.7 (6.5) | 15.8 (6.0) |
| *Missing, n* | *83* |  | *17* | *66* |
| Happiness – Independent (range: 0-6) | 4.8 (1.1) |  | 4.7 (1.1) | 4.9 (1.1) |
| *Missing, n* | *2* |  | *0* | *2* |
| Happiness – Interdependent (range: 1-5) | 4.0 (0.9) |  | 3.8 (0.8) | 4.2 (0.9) |
| *Missing, n* | *106* |  | *25* | *81* |
| Inclusion of community in self (range: 1-6) | 4.0 (1.4) |  | 3.8 (1.2) | 4.1 (1.5) |
| *Missing* | *93* |  | *22* | *71* |
| Sense of belonging (range: 1-4) | 0.5 (0.8) |  | 0.4 (0.7) | 0.5 (0.9) |
| *Missing, n* | *0* |  | *0* | *0* |

 Individuals missing Veggie Meter scores at baseline are due to Veggie Meter operational errors

**Supplementary Table 3.** Full solution of fixed effects from fully adjusted mixed effects linear regression model comparing Veggie Meter scores among Harvest Share and Comparison Community participants

|  | **β (95% CI)** |
| --- | --- |
| **Intercept** | **278.36 (225.79, 330.93)*** |
| **Time** |  |
| Post | **-27.29 (-36.27, -18.30)*** |
| Pre | *Ref.* |
| **Group** |  |
| CSA | 13.07 (-14.14, 40.28) |
| Comparison Community | *Ref.* |
| **Time x Group** |  |
| CSA post | **19.86 (3.33, 36.38)*** |
| Comparison Community post | *Ref.* |
| CSA pre | *Ref.* |
| Comparison Community pre | *Ref.* |
| **Age** | 0.53 (-0.23, 1.28) |
| **Gender** |  |
| Man | -13.03 (-37.93, 11.87) |
| Other | 26.49 (-53.46, 106.44) |
| Woman | *Ref.* |
| **Race** |  |
| Latine | **-63.94 (-113.30, -14.57)*** |
| Non-Hispanic White | -0.44 (-45.25, 44.36) |
| Multiracial | 2.60 (-89.02, 94.21) |
| Another race or ethnicity | -14.08 (-63.80, 35.65) |
| Asian | *Ref.* |
| **Nativity** |  |
| Born in US | -31.01 (-62.93, 0.92) |
| Born outside of US | *Ref.* |
| **Education** |  |
| <High school | 0.41 (-38.93, 39.75) |
| Some high school | -9.79 (-48.5, 28.92) |
| High school or equivalent | -14.69 (-48.21, 18.83) |
| ≥College | *Ref.* |
| **Income** |  |
| <$12,000 | 23.68 (-4.30, 51.66) |
| $12,000-$34,999 | 21.08 (-9.43, 51.60) |
| $35,000-$49,999 | 25.32 (-13.85, 64.49) |
| $50,000-$99,999 | 29.69 (-10.46, 69.83) |
| ≥$100,000 | 16.37 (-27.50, 60.23) |
| Unknown/missing | *Ref.* |
| **Household size** |  |
| 1 person | -10.59 (-45.05, 23.87) |
| 2 persons | 12.45 (-13.16, 38.07) |
| ≥3 persons | *Ref.* |
| **Language preference** |  |
| Chinese | 22.91 (-18.03, 63.85) |
| Spanish | **96.41 (46.30, 146.51)*** |
| English | *Ref.* |

*p<0.05

CSA, Community Supported Agriculture; US, United States

 “Another race or ethnicity” includes the following self-select groups: American Indian/Alaskan Native, Non-Hispanic Black, Southwest Asian and North African, Native Hawaiian and Pacific Islander

High school or equivalent includes high school, Associate’s degree, technical school, and vocational school

**Supplementary Table 4.** Full solution of fixed effects from fully adjusted mixed effects linear regression model comparing number of fruits and vegetables consumed in the last month (26-item) among Harvest Share and Comparison Community participants

|  | **β (95% CI)** |
| --- | --- |
| **Intercept** | **12.82 (10.57, 15.08)*** |
| **Time** |  |
| Post | 0.05 (-0.48, 0.57) |
| Pre | *Ref.* |
| **Group** |  |
| CSA | -0.19 (-1.38, 1.01) |
| Comparison Community | *Ref.* |
| **Time x Group** |  |
| CSA post | **3.69 (2.73, 4.64)*** |
| Comparison Community post | *Ref.* |
| CSA pre | *Ref.* |
| Comparison Community pre | *Ref.* |
| **Age** | **0.08 (0.05, 0.12)*** |
| **Gender** |  |
| Man | -0.72 (-1.79, 0.34) |
| Other | -1.76 (-5.19, 1.67) |
| Woman | *Ref.* |
| **Race** |  |
| Latine | **-2.79 (-4.97, -0.60)*** |
| Non-Hispanic White | -0.48 (-2.38, 1.41) |
| Multiracial | **-6.37 (-10.28, -2.47)*** |
| Another race or ethnicity | -0.38 (-2.56, 1.79) |
| Asian | *Ref.* |
| **Nativity** |  |
| Born in US | **-1.65 (-3.04, -0.26)*** |
| Born outside of US | *Ref.* |
| **Education** |  |
| <High school | **2.80 (1.12, 4.48)*** |
| Some high school | **1.80 (0.15, 3.46)*** |
| High school or equivalent | 0.34 (-1.09, 1.78) |
| ≥College | *Ref.* |
| **Income** |  |
| <$12,000 | 0.72 (-0.49, 1.93) |
| $12,000-$34,999 | 0.29 (-1.04, 1.62) |
| $35,000-$49,999 | 0.84 (-0.86, 2.54) |
| $50,000-$99,999 | -0.64 (-2.36, 1.08) |
| ≥$100,000 | 0.26 (-1.60, 2.12) |
| Unknown/missing | *Ref.* |
| **Household size** |  |
| 1 person | -0.96 (-2.42, 0.51) |
| 2 persons | -0.44 (-1.54, 0.65) |
| ≥3 persons | *Ref.* |
| **Language preference** |  |
| Chinese | -0.18 (-1.93, 1.57) |
| Spanish | **2.69 (0.47, 4.92)*** |
| English | *Ref.* |

*p<0.05

CSA, Community Supported Agriculture; US, United States

 “Another race or ethnicity” includes the following self-select groups: American Indian/Alaskan Native, Non-Hispanic Black, Southwest Asian and North African, Native Hawaiian and Pacific Islander

High school or equivalent includes high school, Associate’s degree, technical school, and vocational school

**Supplementary Table 5.** Full solution of fixed effects from fully adjusted mixed effects linear regression model comparing number of vegetables individuals do not know how to prepare among Harvest Share and Comparison Community participants

|  | **β (95% CI)** |
| --- | --- |
| **Intercept** | **3.42 (1.74, 5.10)*** |
| **Time** |  |
| Post | -0.13 (-0.57, 0.32) |
| Pre | *Ref.* |
| **Group** |  |
| CSA | 0.66 (-0.26, 1.58) |
| Comparison Community | *Ref.* |
| **Time x Group** |  |
| CSA post | **-1.48 (-2.28, -0.67)*** |
| Comparison Community post | *Ref.* |
| CSA pre | *Ref.* |
| Comparison Community pre | *Ref.* |
| **Age** | **-0.02 (-0.05, 0.00)*** |
| **Gender** |  |
| Man | 0.49 (-0.31, 1.29) |
| Other | 0.72 (-1.86, 3.30) |
| Woman | *Ref.* |
| **Race** |  |
| Latine | **2.60 (1.02, 4.18)*** |
| Non-Hispanic White | 0.45 (-0.96, 1.86) |
| Multiracial | 2.46 (-0.48, 5.41) |
| Another race or ethnicity | 1.22 (-0.39, 2.84) |
| Asian | *Ref.* |
| **Nativity** |  |
| Born in US | **1.20 (0.15, 2.24)*** |
| Born outside of US | *Ref.* |
| **Education** |  |
| <High school | 0.58 (-0.66, 1.83) |
| Some high school | 0.30 (-0.94, 1.53) |
| High school or equivalent | 0.37 (-0.70, 1.43) |
| ≥College | *Ref.* |
| **Income** |  |
| <$12,000 | -0.65 (-1.56, 0.26) |
| $12,000-$34,999 | -0.69 (-1.69, 0.31) |
| $35,000-$49,999 | 0.18 (-1.09, 1.44) |
| $50,000-$99,999 | -0.66 (-1.96, 0.63) |
| ≥$100,000 | -0.19 (-1.58, 1.21) |
| Unknown/missing | *Ref.* |
| **Household size** |  |
| 1 person | -0.34 (-1.44, 0.75) |
| 2 persons | -0.12 (-0.94, 0.69) |
| ≥3 persons | *Ref.* |
| **Language preference** |  |
| Chinese | 0.41 (-0.90, 1.71) |
| Spanish | **-2.29 (-3.91, -0.68)*** |
| English | *Ref.* |

*p<0.05

CSA, Community Supported Agriculture; U.S., United States

 “Another race or ethnicity” includes the following self-select groups: American Indian/Alaskan Native, Non-Hispanic Black, Southwest Asian and North African, Native Hawaiian and Pacific Islander

High school or equivalent includes high school, Associate’s degree, technical school, and vocational school

**Supplementary Table 6.** Implementation feedback on the Harvest Share CSA program

| **Theme** | **Key findings** | **Sample quotes** |
| --- | --- | --- |
| Pick-up logistics | - Accessing the rooftop farm for pick-up was generally easy, with participants walking, taking the subway, or driving to the location - Participants appreciated being able to designate alternate pick-up personnel - Suggestion: Offer additional pick-up locations, hours, or days to address scheduling or locale difficulties and to serve additional communities | *“It was pretty easy…After a while my sister would go and pick it up for me, which also was easy because it was easy to ask someone else to pick it up.” (*English-speaking, medium income)  *“That it's every week and that it's only that day and at that time, [there should be] more accessibility to pick up like two different ways, at least if we couldn't go Tuesday, we could go Friday.”* (Spanish-speaking, low income)  *“[…] I think, made it kinda feel a bit like a chore every week because it took, I don’t know, an extra like 30 minutes of our time each week that we had to dedicate and go and pick it up.”* *(*English-speaking, Chinese identifying, high income) |
| CSA staffing | - Friendliness and helpfulness of the CSA staff made pick-up enjoyable | *“I figured it was just gonna be a drive-by, they throw a bag at you and then you leave. It was more like, “Come, stay, talk, farm, do things, learn about it, pick your own party.” Everyone’s really friendly. So, I think it was definitely pretty cool.”* (English-speaking, Chinese identifying, high income)  *“I really like that the people who are there, even when they don’t speak Spanish, treat you like, “I don’t speak much Spanish, but I can help you” … So, the people are very kind to all the people who are there to get their vegetables.”* (Spanish-speaking, low income)  *“It was really nice to go to the farm each week, to kind of engage with people running the Brooklyn Grange there—Celine and her team that was running the program, and the other people in the program…It was really nice even just to have that super quick second, just say, “Hey. How’s it going?” with people that are in neighborhood, in community. We know our upstairs neighbors well. But apart from that, we don’t really know anyone else on this street.”* (English-speaking, high income) |
| CSA set-up | - Participants enjoyed selecting which produce they wanted from the market-style layout - Perceptions of the amount of produce varied - Participants appreciated the types of produce offered but sometimes reported lack of variety across weeks - Suggestions:   - Consider pre-packaged produce as supply was sometimes low at the end of the pick-up window   - Reduce vegetable repetition across weeks   - Include fruit in CSA program | *“It was great. The nice thing about it was that I guess in some ways it didn't feel like it was what I “got” because I actually was choosing it a little bit when I got there. The package wasn't pre-put together, so I think we always took everything. But if there was something that for whatever reason I knew I wouldn't use, I could leave it there.”* (English-speaking, middle income)  *“I would say that some weeks it encompassed the entirety of the vegetable intake. And some weeks it’s more of a supplement. It really depends on what the – the produce that are available. For instance, when they have the Chinese broccoli and then the bok choy and also carrots, that would be the full thing. I just like experimenting with a lot of things like that. On the week that there’s maybe herbs or peppers, then that will be more of a supplement.”* (English-speaking, Chinese identifying, high income)  *“I know that they gave us menu cards or recipe cards, but that was kinda of a very specific recipe. And so, if we were receiving the same thing multiple times, I don’t know, there were just certain things. There was like this one succulent thing that we just had no idea how to use and we didn’t really like very much, the flavor of it, and we got that a handful of times.”* (English-speaking, Chinese identifying, high income) |
| CSA tailoring | - Participants enjoyed that the vegetable types were consistent with their dietary preferences - Produce offerings strengthened participants’ connections to their culture, either for themselves or their children - Unfamiliar vegetables offered an opportunity for learning | *“[Receiving Mexican produce is important] because we already know the flavor and we already know how to cook them more than anything and … because they are fresh, freshly grown, they have better nutrients to buy them here than in the supermarkets.”* (Spanish-speaking, low income)  *“Our family is mixed … So, I think it’s a great introduction to our children, because what we do have access to is the normal vegetables, the broccoli, the tomatoes. Which is great in itself. But there’s even vegetables that I didn’t know that I could cook with, that are Chinese vegetables. So, it’s not only a great introduction to them but to me as well.”* (English-speaking, Chinese identifying, high income)  *“My husband does a lot of the cooking, and I kinda took on figuring out some of these greens. So, now I’m kinda the go-to when it comes to meals that need Chinese greens. ‘Oh, you know how to do this. Can you do this?’”* (English-speaking, high income) |
| Other program activities | - Participants enjoyed the option of participating in other Harvest Share programmatic activities, including workshops and farm tours - Suggestions:   - Clarify if programs are geared towards families with children only   - Add additional workshop locations | *“I think there were other events that came up at Brooklyn Grange that we were invited to that was really lovely. I think there was a movie one night. And then other events on the days that we did pick-ups which were just—it felt like an opportunity to get more connected in the community.”* (English-speaking, middle income)  *“And then there was one dumpling making workshop that we could go to […] That’s something, unfortunately, I haven’t done since the passing of our older family members. So, that’s something that kids would experience […] it was great to have [my child] exposed it while they’re younger.”* (English-speaking, Chinese identifying, high income)  *“I think having the farm as the heart of it was definitely great. I think kind of continuing—there’s definitely at least one [event] that was not held at the farm. But kind of continuing to maybe engage with some of the other places in the community. Whether it’s a coffee shop or specialty store or a restaurant or something like that—maybe having it such that an event is planned there or a meet up is planned there or something like that to kind of add to the locations that are our forms for engagement.”* (English-speaking, high income) |

**Relating Supplementary Table 6 findings to existing literature:**

At its inception, Harvest Share incorporated feedback from our own formative work^1,2^ and that of others to improve engagement and retention in the program, including addressing some of the following previously identified barriers. Participants indicated that the program was well-organized and staffed by welcoming individuals, providing a means of social connection that has previously facilitated engagement^3^ and addressing previous barriers including pickup disorganization^4^. Participants generally indicated that the type and amount of food they received each week was well-aligned with dietary preferences and sufficient in quantity, though some felt the quantity was inadequate some weeks or that the amount left at the end of the pickup window lacked variety, as has been noted in previous programs^4^. While a variety of share sizes were not offered in Growing Season 1, other programs have noted this can improve engagement.^5,6^ Lastly, the pick-up location was generally viewed as convenient—thus facilitating engagement^5,7^—with many participants noting that they now view the pickup location as a staple in their community.

**References**

1. Chan, S. W. C. *et al.* A Culturally Specific Community Supported Agriculture (CSA) Program to Improve Diet in Immigrant Communities in Brooklyn, New York. *Health Promot. Pract.* **26**, 243–248 (2025).

2. Lu, I. *et al.* A qualitative study to inform the development of a subsidized community-supported agriculture program for Chinese Americans in Brooklyn, New York, U.S. *Prev. Med. Rep.* **36**, 102480 (2023).

3. Izumi, B. T. *et al.* Feasibility of Using a Community-Supported Agriculture Program to Increase Access to and Intake of Vegetables among Federally Qualified Health Center Patients. *J. Nutr. Educ. Behav.* **50**, 289-296.e1 (2018).

4. White, M. J. *et al.* The perceived influence of cost-offset community-supported agriculture on food access among low-income families. *Public Health Nutr.* **21**, 2866–2874 (2018).

5. Hanson, K. L., Concepcion, C. & Volpe, L. C. Factors Associated with Participation in Community Supported Agriculture (CSA) among Low-Income Households: A Scoping Review. *Nutrients* **16**, 2450 (2024).

6. Hanson, K. L. *et al.* Participation in cost-offset community-supported agriculture by low-income households in the USA is associated with community characteristics and operational practices. *Public Health Nutr.* **25**, 2277–2287 (2022).

7. Garrity, K. *et al.* Local Food System Approaches to Address Food and Nutrition Security among Low-Income Populations: A Systematic Review. *Adv. Nutr. Bethesda Md* **15**, 100156 (2024).
